# Mitophagy in Doxorubicin-Induced Cardiotoxicity: Insights into Molecular Biology and Novel Therapeutic Strategies

**DOI:** 10.3390/biom14121614

**Published:** 2024-12-17

**Authors:** Heng Zhang, Saiyang Xie, Wei Deng

**Affiliations:** 1 Department of Cardiology, Renmin Hospital of Wuhan University, Wuhan 430060, China; hengzhang98@whu.edu.cn (H.Z.); yangsai1995@whu.edu.cn (S.X.); 2 Hubei Key Laboratory of Metabolism and Related Chronic Diseases, Wuhan 430060, China

**Keywords:** doxorubicin, cardiotoxicity, mitochondria, mitophagy, treatment

## Abstract

Doxorubicin is a chemotherapeutic drug utilized for solid tumors and hematologic malignancies, but its clinical application is hampered by life-threatening cardiotoxicity, including cardiac dilation and heart failure. Mitophagy, a cargo-specific form of autophagy, is specifically used to eliminate damaged mitochondria in autophagosomes through hydrolytic degradation following fusion with lysosomes. Recent advances have unveiled a major role for defective mitophagy in the etiology of DOX-induced cardiotoxicity. Moreover, specific interventions targeting this mechanism to preserve mitochondrial function have emerged as potential therapeutic strategies to attenuate DOX-induced cardiotoxicity. However, clinical translation is challenging because of the unclear mechanisms of action and the potential for pharmacological adverse effects. This review aims to offer fresh perspectives on the role of mitophagy in the development of DOX-induced cardiotoxicity and investigate potential therapeutic strategies that focus on this mechanism to improve clinical management.

## 1. Introduction

Doxorubicin (DOX) is a broad-spectrum antitumor drug widely used in clinical practice. Its clinical application is extensive and holds significant importance, particularly in the following domains. DOX emerges as a cornerstone medication in the therapeutic repertoire for acute leukemia, exhibiting remarkable efficacy in addressing both acute lymphoblastic leukemia and acute myelogenous (granulocytic) leukemia [1]. Often serving as the foundation of treatment protocols, it becomes an invaluable second-line therapy when initial drug choices demonstrate resistance [2], thereby expanding the array of potential therapeutic interventions. DOX also demonstrates exceptional efficacy in the management of both non-Hodgkin’s lymphoma and Hodgkin’s lymphoma, functioning either as the primary therapeutic agent or as a critical component within a multidrug regimen [3,4]. Its potent antitumor activity underscores its importance in the treatment paradigm of malignant lymphomas. Breast cancer represents another key therapeutic indication for DOX [5]. In its treatment strategy, DOX is frequently administered in combination with drugs such as cyclophosphamide, paclitaxel, or other DOX-based regimens to enhance therapeutic efficacy [6,7]. This synergistic combination has become a fundamental aspect of the standard of care for breast cancer treatment. Beyond these malignancies, DOX boasts a versatile application in the treatment of bladder cancer, head and neck cancers, testicular cancer, liver cancer, stomach cancer, and numerous other tumor types [8,9,10,11,12,13,14]. Its broad-spectrum antitumor properties emphasize its pivotal role in the therapeutic repertoire against a diverse array of cancers.

Why does DOX occupy such a pivotal role in clinical practice? Firstly, its remarkable efficacy spans a wide range of tumor types, rendering it invaluable in clinical settings due to its broad-spectrum antitumor capabilities. Its therapeutic prowess is evident in both benign and malignant tumors, underscoring its versatility and significance in clinical applications. Secondly, its potent antitumor activity is a key factor contributing to its effectiveness. By interfering with the chemical structure of DNA and inhibiting nucleic acid synthesis, DOX eliminates tumor cells at a deeper level [15,16]. This potent antitumor activity enables DOX to excel in the treatment of a diverse array of refractory tumors. Furthermore, DOX is highly favored as a component in combination therapy regimens. It holds an indispensable position in numerous combination chemotherapy protocols, where its synergistic interaction with other anticancer agents significantly enhances therapeutic efficacy and mitigates drug resistance [17,18]. Consequently, this contributes to the prolongation of patient survival. In conclusion, as a broad-spectrum antitumor agent, DOX embodies immense value and significance in clinical practice. Its extensive applicability and outstanding efficacy have served as a beacon of hope and provided relief to numerous tumor patients.

**Figure 1:**
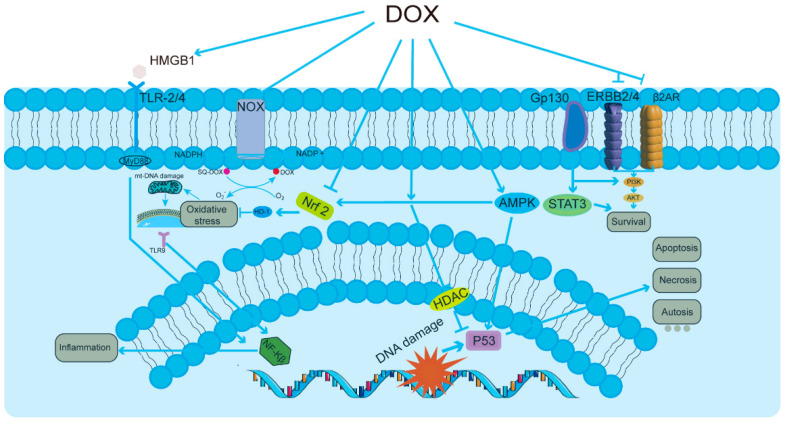
Overview of DOX-induced cardiotoxicity in cardiomyocytes. DOX treatment initiates a complex interplay of multi-focal signaling pathways that play critical roles in cellular responses. The HMGB1/TLRs/NF-κB signaling pathway is activated, leading to enhanced inflammatory responses, while the NOX/Nrf2/HO-1/ROS pathway contributes to oxidative stress by generating reactive oxygen species. Additionally, the AMPK/P53 pathway can induce cell cycle arrest and apoptosis, and the PI3K/AKT/STAT3 pathway is involved in survival signaling. Collectively, these interconnected events result in oxidative stress, mitochondrial DNA dysfunction, increased inflammation, and several forms of cell death, including apoptosis, necrosis, and autosis, ultimately impacting tissue homeostasis and function.

Nonetheless, a major challenge in the clinical application of DOX lies in its dose-dependent cardiotoxicity, posing a formidable limitation on its utilization [19,20,21]. One of the primary characteristics of doxorubicin-induced cardiotoxicity (DIC) (Figure 1) is the initiation of cellular responses in cardiomyocytes [22,23,24], which may lead to congestive heart failure. Among these pathways, the activation of the HMGB1/TLRs/NF-κB signaling pathway leads to heightened inflammatory responses. In parallel, the NOX/Nrf2/HO-1/ROS pathway plays a significant role in generating reactive oxygen species, thereby contributing to oxidative stress. Additionally, the AMPK/P53 pathway is responsible for inducing cell cycle arrest and apoptosis, while the PI3K/AKT/STAT3 pathway facilitates survival signaling. Together, these interrelated processes result in oxidative stress, mitochondrial DNA dysfunction, increased inflammation, and various forms of cell death, including apoptosis, necrosis, and autophagic cell death (autosis), ultimately affecting tissue homeostasis and function, as shown in Figure 1. This adverse effect not only diminishes the quality of life for cancer patients but also significantly increases the risk of mortality. Among these deleterious effects, mitochondrial damage stands out as particularly pronounced [25,26]. DOX first accumulates in the mitochondria of cardiomyocytes, where it binds to the phospholipid cardiolipin in the inner mitochondrial membrane, blocking the electron transport chain and inhibiting the function of complexes I and II, leading to a significant increase in reactive oxygen species (ROS). Excessive production of ROS disrupts the mitochondrial membrane lipids, which further leads to mitochondrial dysfunction and cell death [27,28]. DOX binds to DNA and topoisomerase 2β (Top2β) to form the Top2–DOX–DNA cleavage complex, which triggers cell death [29]. The inhibition of Top2β, an enzyme specific to cardiac mitochondria, leads to further disruption of mitochondrial structure and function [30]. Moreover, a novel type of cell death, “iron death” (Ferroptosis), is also involved in DIC [31]. DOX upregulates heme oxygenase 1, releasing free iron and forming an active iron–DOX complex, which further promotes the oxidation of mitochondrial membrane lipids, leading to cell death [32]. Autophagy is an important mechanism by which cells remove damaged mitochondria and maintain homeostasis [33]. DOX affects the PI3Kγ pathway and activates the PI3Kγ/Akt/mTOR cascade by inhibiting autophagic flux and suppressing the autophagy initiation process, thus further exacerbating the impairment of mitochondrial function [34]. DOX-mediated ROS production and lipid peroxidation can alter the activity of membrane proteins, such as mitochondrial calcium channels, thereby affecting Ca^2+^ homeostasis and signaling [35,36]. In addition, DOX impairs the expression and activity of myocardial sarcoplasmic reticulum ryanodine receptor 2 (RyR2) and sarcoplasmic reticulum Ca^2+^-ATPase (SERCA2), major participants in myocardial contraction, further reducing myocardial contractility [37,38]. In conclusion, the primary features of DOX’s dose-dependent cardiotoxicity include the impairment of cardiac mitochondrial function, which involves several facets of ROS production, the inhibition of Top2β, induction of iron death, the inhibition of autophagic flux, and the disturbance of Ca^2+^ homeostasis. Together, these interrelated pathways cause damage to cardiomyocytes and a reduction in heart function.

However, there is no systematic overview of the various modes of action of the mitophagy mechanism in DIC so far, nor is there a comprehensive assessment of the drugs and potential molecular targets that can be used to treat DIC with therapeutic efficacy from the perspective of mitophagy. Therefore, studies exploring the role of mitophagy in DIC are important for understanding its pathogenesis and developing effective treatments.

## 2. The Basic Concept and Function of Mitophagy

Mitophagy represents a form of selective cellular autophagy that involves the removal of dysfunctional mitochondria from the cell through autophagic mechanisms [39]. Ubiquitin-dependent mitophagy encompasses mechanisms such as PTEN-induced kinase 1 (PINK1)/Parkin-mediated and Parkin-independent [40].Conversely, ubiquitin-independent mitophagy is characterized by mitophagy receptor-mediated pathways [40].

### 2.1. Major Pathways Involved in Mitophagy

**Figure 2:**
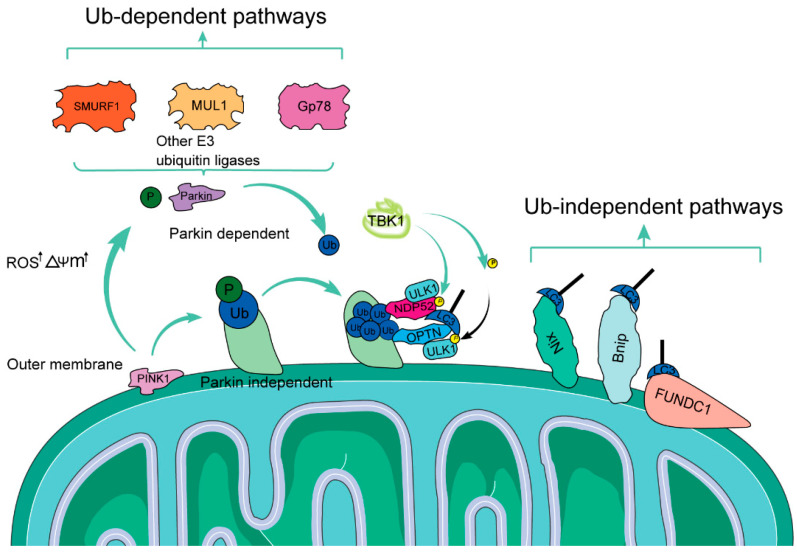
Landscape of the mitophagy mechanisms. Mitochondrial autophagy involves multiple interconnected mechanisms that can be broadly categorized into ubiquitin (Ub)-dependent and ubiquitin-independent pathways. The PINK1/Parkin pathway is the most well-studied ubiquitin-dependent mechanism. In addition, a group of mitochondrial autophagy receptors, such as Nix, Bnip3, and FUND1, can directly bind to LC3 without requiring extensive ubiquitination, which characterizes the ubiquitin-independent pathway. These distinct yet complementary pathways play crucial roles in regulating mitochondrial quality control and cellular homeostasis.

The mechanisms of mitophagy can be broadly divided into two broad categories: ubiquitin-dependent pathways and ubiquitin-independent pathways, as shown in Figure 2 [41]. The mechanisms of mitochondrial autophagy can be broadly categorized into two main types: ubiquitin-dependent pathways and ubiquitin-independent pathways. Ubiquitin-dependent mitochondrial autophagy includes mechanisms such as the PTEN-induced kinase 1 (PINK1)/Parkin-mediated pathway, as well as Parkin-independent processes. In contrast, ubiquitin-independent mitochondrial autophagy is characterized by mitochondrial autophagy receptors, such as Nix, Bnip3, and FUND1, which directly interact with LC3 without relying on ubiquitination (Figure 2).

#### 2.1.1. Ubiquitin-Dependent Pathway-Mediated Mitophagy: PINK1/Parkin

PINK1/Parkin stands as a notable example of Ub-dependent pathways. PINK1 is a serine/threonine-containing mitochondrial targeting sequence acid kinase that can target damaged mitochondria to initiate mitochondrial autophagy to protect cellular functions [42]. In normal mitochondria, PINK1 is transported to the inner mitochondrial membrane, cleaved, and then degraded by the ubiquitin-protease system [43]. However, when mitochondria are depolarized or damaged, PINK1 accumulates in large amounts on the outer mitochondrial membrane (OMM), recruits Parkin to damaged mitochondria, and then activates Parkin by upregulating the level of self-phosphorylation and phosphorylating ubiquitin on OMM [44]. After activation, Parkin not only recognizes damaged mitochondria but also ubiquitinates a large number of proteins on OMMs, resulting in substrate ubiquitin chains [45]. PINK1 and Parkin work in tandem to regulate mitophagy, ensuring mitochondrial quality.

#### 2.1.2. Parkin-Independent Ubiquitin-Dependent Pathway

Moreover, beyond the PINK1–Parkin pathway, there exist non-Parkin-dependent ubiquitin-dependent pathways. Receptor proteins like NIX, BNIP3, and FUNDC1 can be directly recruited to mitochondria by phosphorylated ubiquitin facilitated by PINK1. These receptors then attract LC3, enabling autophagosomes to engulf mitochondria. In contrast to the ubiquitination process driven by PINK1/Parkin, numerous proteins containing LIR motifs on the outer mitochondrial membrane (OMM) serve as autophagy receptors [46,47]. They can engage directly with LC3 without ubiquitination, initiating mitophagy. Representative pathways include BNIP3L/NIX and FUNDC1. A common trait among these pathways is the presence of LC3 binding sites, which facilitates the incorporation of mitochondria into autophagic vesicles [48,49]. This process eliminates damaged or unwanted mitochondria, maintaining the stability of mitochondrial structure and function.

#### 2.1.3. The Role of Other E3 Ubiquitin Ligases in Mitophagy

In the intricate process of mitophagy, several E3 ubiquitin ligases play pivotal roles in ubiquitinating mitochondrial proteins, thereby triggering the engulfment and degradation of damaged mitochondria by autophagosomes [50]. Among these, SMURF1, MUL1, and Gp78 are notable E3 ubiquitin ligases that have been implicated in this process [51]. SMURF1, an E3 ubiquitin ligase of the HECT family, regulates diverse processes such as TGF-β signaling, cell cycle progression, and bone formation [52]. It also participates in mitophagy, where it ubiquitinates specific mitochondrial proteins [53]. The precise ubiquitination mechanism remains unclear, but it likely tags proteins for autophagic recognition, initiating mitophagy, and targeting damaged mitochondria for degradation. MUL1, a RING finger E3 ubiquitin ligase at the outer mitochondrial membrane (OMM), features a unique RNF domain with SUMO and ubiquitin ligase activities; it regulates mitochondrial morphology and dynamics via interactions with Drp1 and Mfn2 [54]. In addition, MUL1 ubiquitinates mitochondrial proteins, notably Mfn2, for ubiquitin–proteasome degradation, thereby inducing mitophagy [55]. The precise mechanism of MUL1-mediated mitophagy is still under investigation. Gp78, another E3 ubiquitin ligase, is involved in mitophagy. It ubiquitinates target proteins, potentially mitochondrial ones, marking them for autophagosomal degradation [56,57]. This ubiquitination may signal the autophagic machinery to recognize and engulf damaged mitochondria, initiating mitophagy, though the precise role of Gp78 in this process is not fully understood.

### 2.2. The Main Functions Performed by Mitophagy

#### 2.2.1. Maintains Mitochondrial Homeostasis

Mitophagy serves a crucial role in maintaining the health and functionality of cells by executing two primary functions: the timely removal of damaged mitochondria and the subsequent maintenance of mitochondrial quality [58]. Mitochondria, the “energy factories” within cells, are highly susceptible to disruptions caused by diverse internal and external factors, potentially leading to compromised function [59]. By efficiently eliminating these dysfunctional mitochondria, mitophagy prevents their accumulation and subsequent exacerbation of cellular damage [60]. Furthermore, this process fosters the generation of new, healthy mitochondria, thereby sustaining the overall quality and continuous, stable energy production capabilities of the cellular mitochondria [61]. In essence, mitophagy ensures that cells are equipped with optimal energy-producing machinery, critical for their survival and proper functioning.

#### 2.2.2. Prevents Cell Damage

Mitophagy plays a pivotal role in safeguarding cells against harm by mitigating oxidative stress and preventing apoptosis [62]. Specifically, damaged mitochondria often give rise to elevated levels of oxidative stress, marked by an increase in ROS [63]. By promptly eliminating these dysfunctional organelles, mitophagy curtails the production and accumulation of ROS, thereby minimizing the cellular damage inflicted by oxidative stress [64]. Furthermore, damaged mitochondria can also initiate apoptosis, a programmed cell death mechanism [65]. Mitophagy intervenes in this process by rapidly eliminating the damaged mitochondria and lowering the release of signals that trigger apoptosis, protecting cells from dying too soon [66]. In summary, mitophagy effectively guards against cellular injury by counteracting oxidative stress and preventing the onset of apoptosis.

#### 2.2.3. Promotes Cell Survival

Mitophagy also plays a fundamental role in promoting cell survival and vitality by supporting cellular metabolism and enhancing adaptability [67]. By preserving mitochondrial homeostasis and quality, mitophagy enables cells to execute vital metabolic processes necessary for growth, proliferation, and repair [67]. This ensures that cells have the energy and resources to fulfill their biological functions. Additionally, under challenging conditions such as nutrient scarcity and cellular senescence, mitophagy fosters resilience by dynamically adjusting the number and functional status of mitochondria [68]. This adaptability allows cells to better cope with environmental stresses and maintain their survival, highlighting the essential contribution of mitophagy to the overall health and longevity of cells.

In brief, mitophagy plays a key role in maintaining mitochondrial homeostasis, preventing cell damage, and promoting cell survival. It ensures the normal physiological activities and adaptability of cells by selectively eliminating damaged mitochondria, maintaining mitochondrial quality, reducing oxidative stress, and preventing apoptosis. In order to understand the maintenance process of cellular homeostasis as well as the incidence and development of illnesses, it is crucial to research the molecular mechanism and regulatory network of mitophagy.

## 3. The Effects of DOX on Mitophagy

### 3.1. DOX Affects Mitophagy Through the PINK1–Parkin Pathway

Firstly, DOX has the capability to inflict DNA damage, constituting one of its primary mechanisms underlying its potent anticancer effects [5,69]. The crucial tumor suppressor protein p53 becomes activated when cellular DNA is damaged. With this activation, p53 takes center stage in the cellular stress response, directing vital functions like cell cycle regulation, DNA repair pathways, and, in the end, apoptosis [70]. When p53 is activated, it can interact with the E3 ubiquitin ligase Parkin in the cytoplasm, preventing Parkin from moving from the cytosol to the outer membrane of the mitochondria [71]. The PINK1/Parkin-mediated mitophagy pathway is impeded due to the disruption in Parkin’s normal recruitment to the outer mitochondrial membrane [42]. Consequently, damaged mitochondria fail to be efficiently eliminated, leading to their accumulation within the cell and precipitating a cascade of detrimental reactions. One of the key mechanisms of DOX cardiotoxicity is the buildup of damaged mitochondria [72]. These damaged mitochondria not only cannot supply the cells with enough energy but they also leak toxic compounds (like ROS) that worsen apoptosis and damage to the cells [72]. In summary, DOX interferes with the activation of p53 in response to DNA damage, which in turn hinders the translocation of Parkin to the outer mitochondrial membrane. As a result, the PINK1/Parkin-mediated mitochondrial autophagy is also suppressed. Cardiotoxicity is the result of this process, which causes damaged mitochondria to accumulate.

### 3.2. DOX Affects Mitophagy via Bnip3

The mechanism by which DOX triggers Bnip3-mediated mitophagy, leading to mitochondrial permeability transition pore (mPTP) opening and mitochondrial depolarization, is not fully described in the literature [73]. However, we can hypothesize based on the existing knowledge of mitophagy, Bnip3′s role, and the effects of DOX on cells. Bnip3 is a multifunctional protein localized to the outer mitochondrial membrane that induces mitophagy under stress conditions such as hypoxia and nutrient deficiency [74]. It binds to autophagosomal proteins like LC3 through its BH3 domain, mediating the recognition and encapsulation of damaged mitochondria for degradation [75].

DOX, by intercalating into DNA and inducing oxidative stress, leads to mitochondrial damage and ROS production. This damage activates intracellular signaling pathways that increase Bnip3 expression. Bnip3, in turn, interacts with LC3 to form a mitophagy complex, allowing damaged mitochondria to be degraded. During this process, mitochondria may undergo morphological and functional changes, such as mPTP opening and depolarization. The opening of the mPTP allows small molecules to pass freely, lowering mitochondrial membrane potential and causing depolarization. While Bnip3 is likely involved in these processes, the exact mechanism remains unclear. Further investigation is required to fully elucidate how DOX induces mPTP opening and mitochondrial depolarization.

In order to further comprehend the effects of DOX on mitophagy more specifically, it is pertinent to present concrete experimental data that elucidate the regulatory role of DOX on mitophagy and its subsequent impact on cardiomyocytes (Table 1). This summary is crucial for understanding the potential side effects and therapeutic interventions associated with DOX treatment in the context of cardiovascular health.

In this table, we observed an intriguing phenomenon. Despite the model and the identical phenotype, the changes in mitophagy showed significant variation. This could be due to several factors. First, small differences in experimental conditions, such as variations in treatment doses, timing, or cell culture conditions, could impact mitochondrial autophagy. Even slight changes in these parameters can lead to different outcomes. Additionally, cell type or individual variations could also contribute to differences in autophagy responses. Different cell lines or primary cells might activate distinct autophagy pathways in response to stress, which can result in variation in mitochondrial autophagy despite similar phenotypic observations. Moreover, the regulation of mitochondrial autophagy is complex, involving multiple signaling pathways and proteins beyond just Bnip3. Variations in any of these regulatory networks could influence the observed differences in autophagy. Finally, inherent biological variability in experimental data is common, and the observed differences might simply reflect this variability. To address these issues, it is essential to standardize experimental conditions, increase sample sizes, and conduct more replicates. Additionally, performing deeper molecular analyses to assess other key autophagy regulators could help clarify these differences. Expanding the study to include multiple cell models might also provide further insights into whether these variations are specific to certain conditions or are more widely applicable.

## 4. The Pathogenesis of Mitophagy in DIC

### 4.1. Mitochondrial Dysfunction

Mitophagy, a vital cellular mechanism for clearing damaged or surplus mitochondria to sustain functional integrity and cellular homeostasis, is intricately tied to the emergence of cardiotoxicity. When this process is disrupted, it initiates a cascade of detrimental effects that can profoundly impact heart health. Initially, the imbalance in mitochondrial autophagy leads to mitochondrial fragmentation [81], a process where once-cohesive mitochondria undergo morphological changes, becoming smaller and more fragmented. This, in turn, disrupts the efficient production of ATP, the energy currency of the cell, as fragmented mitochondria are less capable of carrying out oxidative phosphorylation [82]. Furthermore, the persistence of damaged mitochondria due to disrupted autophagy fosters an environment conducive to oxidative stress [83]. ROS and other oxidative substances, released from these malfunctioning mitochondria, cause widespread damage to various cellular components, including DNA, proteins, and lipids, further exacerbating mitochondrial dysfunction [84]. These combined consequences ultimately lead to a higher risk of apoptosis, in which badly damaged mitochondria release cytochrome c and other apoptotic signals, which initiate programmed cell death [85]. The loss of cardiac cells due to apoptosis leads to reduced heart function and contributes to the progression of heart failure. Essentially, the disruption of mitophagy plays a crucial role in the onset of cardiotoxicity, initiating a cascade of events that begins with mitochondrial fragmentation and ATP depletion, advances through oxidative stress, and ultimately results in cell death. These processes significantly impair the heart’s normal function.

### 4.2. Changes in the Structure and Function of the Heart

The relationship between mitochondrial dysfunction and mitophagy is often bidirectional, with each process influencing the other. Impaired mitophagy can lead to the accumulation of damaged mitochondria, exacerbating mitochondrial dysfunction. Conversely, dysfunctional mitochondria may disrupt normal mitophagic processes, highlighting their critical interdependence in maintaining cellular health and preventing disease [86]. The initial consequence is a decrease in ATP production, resulting in an inadequate supply of energy for cardiomyocytes [87]. This energy insufficiency adversely affects the function of ion channels in cardiomyocyte membranes, particularly ATP-sensitive potassium channels (sarcKATP), which are sensitive to cellular energy states [88]. Concurrently, mitochondrial dysfunction significantly elevates the production of ROS, which damages proteins, lipids, and DNA within cardiomyocytes. This damage further disrupts the function of ion channels and transporters, thereby increasing the risk of arrhythmias [89]. Mitochondrial dysfunction significantly disrupts the balance of calcium ion uptake and release, which in turn affects the electrical activity and contractile function of cardiomyocytes [90]. This disruption is a critical factor contributing to the development of arrhythmias. Furthermore, the oxidative stress and insufficient energy supply associated with mitochondrial dysfunction can lead to cardiomyocyte apoptosis [91]. As these cells continue to die, the ventricular walls thicken and the ventricular cavity expands, resulting in ventricular dilation. The increase in wall thickness occurs as a compensatory response to the death of cardiomyocytes. The remaining healthy myocytes undergo hypertrophy, increasing in size to help maintain the heart’s pumping ability. Additionally, structural changes such as fibrosis may occur over time, further contributing to wall thickening. Overall, these changes indicate that the heart is working to adapt to the reduction in cells while maintaining its function [92]. This dilation is often accompanied by myocardial remodeling, an adaptive response of the heart to chronic stress or volume overload [93]. Myocardial remodeling includes processes such as cardiomyocyte hypertrophy and interstitial fibrosis. However, the remodeling process further compromises the function and structure of the heart muscle, leading to increased ventricular dilation [94]. As this dilation and myocardial remodeling progress, both systolic and diastolic functions of the heart decline progressively, rendering it unable to meet the body’s physiological needs and ultimately resulting in heart failure [95]. In response to this condition, the body activates a series of neurohumoral regulatory mechanisms to maintain short-term circulatory stability [96]. Unfortunately, these mechanisms can have detrimental long-term effects, increasing the burden on the heart and exacerbating myocardial damage. This creates a vicious cycle that further deteriorates cardiac health.

## 5. Therapeutic Strategies That Target Mitophagy

### 5.1. Pharmaceutical Drugs

#### 5.1.1. Vericiguat

Vericiguat is a soluble guanylate cyclase (sGC) stimulator that has been widely used in the treatment of patients with heart failure with reduced ejection fraction (HFrEF) since its approval in China in May 2022 [97,98]. Vericiguat upregulates the expression of protein kinase G1 (PRKG1), which in turn activates PINK1 [99]. Vericiguat promotes the recognition and clearance of damaged mitochondria by activating PINK1, thereby alleviating the damage to cardiomyocytes caused by mitochondrial dysfunction [99]. Damaged mitochondria are often accompanied by the leakage of mitochondrial DNA (mtDNA), which leads to the accumulation of DNA fragments in the cytoplasm, which in turn triggers an inflammatory response and cell damage [100]. Vericiguat reduces mtDNA leakage by regulating mitophagy, thereby inhibiting the occurrence of inflammatory responses and cell damage. Vericiguat also inhibits the activity of the interferon gene stimulating protein (STING)/ interferon regulator 3 (IRF3) signaling pathway [99], which plays an important role in the inflammatory response, and its overactivation can lead to cardiomyocyte damage. By inhibiting this signaling pathway, vericiguat helps reduce inflammation in heart tissue and protects cardiomyocytes from damage.

#### 5.1.2. Metformin

The antidiabetic drug metformin has been shown to reduce cardiac damage in various pathological conditions, including cardiotoxicity induced by the anticancer DOX [101,102]. Van et al. reported that metformin significantly suppressed the accumulation of LC3-II levels in both total cell lysates and mitochondrial fractions [103]. Furthermore, their study revealed that metformin also reduced the elevation of LC3-II levels induced by the lysosomal protease inhibitors, pepstatin A and E64d, in both total cell lysates and mitochondrial fractions [103]. These findings indicate that metformin effectively inhibits DOX-induced mitophagy as well as the subsequent death of cardiomyocytes. The cardioprotective effect of metformin against DOX cardiotoxicity appears to be closely linked to its ability to modulate mitophagy [103]. By preventing excessive degradation of mitochondrial components, metformin helps maintain mitochondrial integrity, which is crucial for cellular energy production and overall cardiac function. Future studies should explore the precise molecular pathways through which metformin exerts these effects, potentially leading to innovative therapeutic strategies for mitigating chemotherapy-induced cardiotoxicity.

#### 5.1.3. Neuraminidase 1 Inhibitors

Neuraminidases (NEUs), also known as sialidases, are a family of glycosidases responsible for the removal of terminal sialic acid from glycoproteins and glycolipids [104]. Different genes have encoded four types of NEUs (NEU1, NEU2, NEU3, and NEU4) in mammals, and they have been identified based on their distinct enzymatic properties and subcellular location [105]. NEU1 is highly expressed in the heart and plays a role in multiple cardiovascular disorders, such as coronary heart disease, ischemia–reperfusion injury, myocardial hypertrophy, and heart failure [106,107,108]. Oseltamivir (OSE) is a NEU1 inhibitor. According to the findings of one study, rats treated with DOX in addition to OSE demonstrated retained heart function and defense against DOX-induced cardiomyopathy. Further research has revealed that the beneficial effects of OSE are associated with the suppression of dynamin-related protein 1 (Drp1)-dependent mitophagy. The elevated NEU1 boosts the expression of Drp1, subsequently enhancing mitochondrial fission and triggering PINK1/Parkin pathway-mediated mitophagy, ultimately creating a detrimental feedback loop towards myocardial apoptosis and cell death [109]. OSE administration selectively restrains the upsurge of NEU1 in cardiomyocytes subjected to DOX, leading to decreased Drp1 expression, the suppression of PINK1 stabilization on mitochondria, and the inhibition of Parkin translocation to mitochondria. This ultimately alleviates excessive mitochondrial fission and mitophagy, thereby mitigating the subsequent progression of cellular apoptotic processes [109]. The study revealed that NEU1 plays a crucial role in enhancing the effects of DOX by facilitating drp1-mediated mitochondrial fission and mitophagy, unveiling that NEU1 inhibitors could be a potential strategy for addressing DIC.

#### 5.1.4. Donepezil

Donepezil, an acetylcholinesterase inhibitor, exerts cardioprotective effects against various heart diseases [110,111,112]. Donepezil reduces Drp-1 phosphorylation by activating the muscarinic acetylcholine receptor (mAChR), which restores cardiac mitochondrial dynamics and reduces excessive mitophagy. This effectively protects the heart against DIC by easing mitophagy overload, as demonstrated by T. Khuanjing et al. in the DIC. Furthermore, in both breast cancer cell lines, donepezil did not lessen the anticancer impact of DOX [113]. The results indicate that donepezil may be one of the potential solutions for preventing cardiotoxicity caused by DOX in chemotherapy patients.

### 5.2. Traditional Medicine

#### 5.2.1. Sphingosylphosphorylcholine

Sphingosylphosphorylcholine (SPC) is a physiologically active sphingolipid in plasma. Previous research has demonstrated that SPC significantly reduces the risk of cardiac cell death brought on by hypoxia [114,115]. Furthermore, SPC could widely regulate mitochondrial function. For instance, SPC can increase the release of mitochondrial cytochrome C in neural 2a cells, which triggers anti-apoptotic autophagy and mitochondria-mediated apoptosis, and lower ROS levels in hypoxic cardiomyocytes [116]. It is worth noting that SPC has a potential pathological significance in regulating mitochondrial function. According to a study’s findings, SPC (2.5 μM) considerably ameliorated DOX-induced pericardial edema, myocardial vacuolization, and apoptosis. Furthermore, in DOX-treated H9c2 cells (1 μM), SPC (2.5 μM) can significantly inhibit apoptosis and stimulate cell proliferation; however, this effect is reliant on the restoration of mitochondrial homeostasis, which includes restored levels of ATP, mitochondrial superoxide, and mitochondrial membrane potential. It was conclusively confirmed that SPC restores mitochondrial homeostasis by alleviating excessive mitophagy induced by DOX. Mechanistically, calmodulin levels were reduced by SPC, which inhibited Parkin activation and Parkin-dependent mitophagy [117]. The findings indicate that reducing DIC by targeting SPC may be a new approach to salvage chemotherapy damage.

#### 5.2.2. Harpagoside

The monocase of *Scropularia ningpoensis*, Harpagoside (HAR), is characterized by its various pharmacological effects, such as anti-inflammatory, neuroprotective, and antioxidant properties, which can be beneficial in neurological diseases like brain cancer and osteoarthritis [118,119]. Li et al. found that HAR significantly improves cardiac function, myocardial structural lesions, and mitophagy flux after DOX administration [79]. Additional data showed that HAR promotes Parkin translocation to mitochondria and substantially restores Parkin-mediated mitophagy by inhibiting the binding of p53 and Parkin. Importantly, the cell viability analysis demonstrated that the cardioprotective validity of HAR did not interfere with the anticancer effect of DOX on MCF-7 and HepG2 cells [79]. The study established a p53-Parkin-mediated cascade of mitophagy deficiency—mitochondrial homeostasis—apoptosis, revealing that HAR exerts cardioprotective benefits against DIC by acting on a novel interaction between p53 and Parkin.

#### 5.2.3. Ellagic Acid

Berries, vegetables, and fruit all naturally contain a molecule called polyphenolic ellagic acid (EA) [120]. EA has been demonstrated to promote cell cycle exit and inhibit the proliferative ability of certain cancer cells [121]. In addition, EA has been proven to decrease oxidative damage in several other diseases, including nephrotoxicity and hypertension [122,123]. Abhinav et al. discovered that EA significantly lowers mitochondrial fission and cell death in cells treated with DOX [124]. Notably, EA similarly suppresses mitochondrial injury and cell death induced by Bnip3 overexpression. Dhingra et al. found a novel signaling axis that functionally links Bnip3 and EA for the control of cardiac cell death. This finding offers strong evidence that EA inhibits Bnip3 activity, hence suppressing mitochondrial damage and necrotic cell death of cardiomyocytes [125]. Based on this finding, we boldly hypothesize that EA could benefit cancer patients receiving anthracycline therapy by inhibiting Bnip3-induced mitochondrial damage and reducing mitophagy, as well as cardiac dysfunction.

#### 5.2.4. Berberine

Berberine (BBR) is an isoquinoline alkaloid that occurs naturally in a wide variety of medicinal plants and has a long history of usage [126]. Numerous studies have demonstrated that BBR possesses a variety of pharmacological properties, including antibacterial, anti-inflammatory, antioxidant, anticancer, neuroprotective, and cardioprotective properties [127]. Zhang et al.’s findings suggest that BBR protects AC16 cells and zebrafish hearts from DIC. Bcl-xL knockdown in AC16 cells and zebrafish demonstrated that Bcl-xL is required for BBR’s anti-apoptotic activity. DOX administration facilitates Beclin1’s bonding with Bcl-xL, resulting in the reversal of mitophagy and increased ROS accumulation in AC16 cells. Mitophagy in AC16 cells and zebrafish hearts is enhanced by BBR pretreatment by separating the Bcl-xL–Beclin1 complex and decreasing ROS accumulation, while the impact of BBR is lessened when mitophagy is inhibited. Intriguingly, BBR increases Bcl-xL binding to Bnip3 and mitophagy, significantly ameliorating DOX-induced cardiac dysfunction in zebrafish, whereas Bcl-xL knockdown abolishes this effect, indicating that Bcl-xL may play a beneficial role in BBR-induced mitophagy [128]. The BBR reaction to DOX was found to exhibit a biphasic dose–response effect, as shown by Zhang et al. When cells were treated with low-dose BBR, cardioprotective qualities were demonstrated (≤1 μM in cells and <10 μM in zebrafish); however, when BBR doses were relatively high, no protective effect was detected [128]. The findings indicate that Bcl-xL is responsible for the protective effects of low-dose BBR against DOX-induced cardiotoxicity, but more research is needed to determine the optimal dose of BBR.

#### 5.2.5. Mesaconine

Fuzi, which is the lateral root of *Aconitum carmichaelii*, has a distinct ability to restore vital energy during resuscitation and has been frequently utilized in clinics [129]. Mesaconine is widely used as a cardiotonic component in Fuzi and has been found to be effective in several cardiomyopathy models [130,131]. Zhou et al. study revealed that mesaconine can have a significant impact on reversing the typical cardiac dysfunction, ectopic myocardial energy disturbance, and impaired mitophagy in cardiomyocytes [95]; the cardioprotective effect of mesaconine is primarily attributed to its ability to promote the restoration of mitophagy in cardiomyocytes, as evidenced by the elevated expression of PINK1, a key mediator of mitophagy induction. The protective effects of mesaconine could be completely eliminated by silencing PINK1 or deactivating mitophagy [95]. Taken together, these findings suggest that the cardioprotective effects of mesaconine appear to be dependent on the activation of PINK1-induced mitophagy and that mesaconine may become a promising therapeutic agent for the treatment of DOX-associated heart failure.

### 5.3. Potential Targets

#### 5.3.1. Rubicon

Run domain Beclin-1-interacting and cysteine-rich domain-containing protein (Rubicon) is an inhibitor of cellular autophagy discovered when the Beclin1-interacting protein was first studied [132]. Rubicon is a component of PI3KC3, a protein essential for controlling the maturation of autophagosomes. It specifically attaches to Uvrag within the PI3KC3 subunit, disrupting the connection between Uvrag and C-Vps, which ultimately hinders the maturation process of autophagosomes [133]. Rubicon binds to Beclin1 and Uvrag to inhibit autophagy pathways as well as phagocytosis [134]. Liu et al. demonstrated that in Rubicon-deficient wild-type mice, the administration of DOX results in cytoplasmic vacuolation and collagen buildup, raises the levels of serum lactate dehydrogenase and cardiac creatine kinase activity, higher levels of ROS, decreases ATP levels, notable damage to mitochondria, and an increase in the thickness of the left ventricular wall. The effects are mitigated by mechanisms that improve autophagy flux, mitophagy, and mitochondrial dynamics [135], indicating that Rubicon could be a promising molecular target for partially preventing and treating DIC.

#### 5.3.2. Mdivi-1

PGC-1α is considered to be a major regulator of mitochondrial biogenesis, and this process is closely associated with mitophagy through the PINK1/Parkin pathway [136]. DOX-induced suppression of PGC-1α and its downstream targets, as well as mitochondrial function-related proteins, is mitigated by inhibiting mitophagy via Mdivi-1 [137]. Additionally, Mdivi-1 was discovered to reduce the activation of the PINK1/Parkin pathway caused by DOX and protect mitochondrial biogenesis [138]. This helps to alleviate the excessive production of mitochondrial superoxide and mitochondrial dysfunction induced by DOX. Moreover, mito-tempo can efficiently attenuate the activation of the PINK1/Parkin pathway and rescue cells from adverse effects caused by DOX [137]. Co-incubation of Mdivi-1 with DOX does not reduce the cytotoxicity of DOX against HL-60 cells [137]. These findings suggest that blocking mitophagy with Mdivi-1 protects the heart from DOX-induced cardiac injury. This highlights mitophagy as a novel therapeutic target to ameliorate DOX-induced cardiotoxicity while preserving its anticancer effects.

#### 5.3.3. MiR-147-y

MiR-147, a microRNA produced when Toll-like receptor stimulation occurs, is responsible for regulating murine macrophage inflammatory responses [139]. In recent research, it was found that the overexpression of miR-147-y in rat cardiomyocytes leads to an improvement in cell damage in rat myocardial infarction models [140]; the study by Gao et al. demonstrated that the cell death caused by DOX was reduced by improving mitophagy, as evidenced by a decrease in P62 levels and an increase in various markers such as LC3, PINK1, Parkin mRNA, LC3II/I, beclin-1, PINK1, and Parkin, including p-parkin (Ser65) protein expression. Additionally, the transfection of a miR-147-y mimic results in the inhibition of cell apoptosis, as indicated by the suppression of caspase-3 transcription and cleaved caspase-3 translation [141]. Nevertheless, when cells are transfected with an miR-147-y inhibitor, it decreases DOX-induced mitophagy and enhances apoptosis [141]. The study provides valuable insights into how miR-147-y helps protect cardiomyocytes from DIC. It shows that miR-147-y is essential for regulating mitophagy in cardiomyocytes, which helps reduce the damage caused by DOX. This research sheds light on the potential therapeutic implications of miR-147-y in mitigating the adverse effects of DOX on cardiomyocytes.

#### 5.3.4. IGF-IIR

IGF-IIRα, a truncated form of IGF-IIR, plays a vital role in cardiac growth and remodeling [142,143,144]. We discovered that IGF-IIRα primarily localizes to mitochondria, leading to increased oxidative stress in these organelles. This stress is significantly worsened by DOX treatment, resulting in a notable disruption in mitochondrial membrane potential and increased release of cytochrome C, which in turn leads to elevated cleaved caspase 3 activity [145]. The overexpression of IGF-IIRα in combination with DOX treatment causes significant disruptions in several key proteins, including p-AMPK, p-ULK1, Parkin, PINK1, LC3, and p62. These disruptions are more severe when IGF-IIRα is overexpressed in conjunction with DOX treatment, suggesting that the overexpression of IGF-IIRα in combination with DOX treatment significantly affects these crucial proteins related to mitophagy [145]. Researchers found that IGF-IIRα leads to mitochondrial oxidative stress and perturbed mitophagy, contributing to DIC [142], which challenge the traditional view that oxidative stress is the primary determinant of the cardiac side effects of DOX, suggesting the involvement of additional mechanisms such as mitochondrial dysfunction, DNA damage, and dysregulation of mitophagy. The study highlights the significant role of IGF-IIRα in disrupting mitochondrial homeostasis and mitophagy. The findings have important implications for future research and the development of treatments for DOX-induced cardiomyopathy. Targeting IGF-IIRα could potentially provide a new approach for mitigating the cardiotoxic effects of DOX, bringing hope for better treatment strategies in the future.

#### 5.3.5. SESN2

It has been demonstrated that the stress-inducible protein sestrin2 (SESN2) protects against DOX-induced cardiomyopathy by regulating mitophagy and mitochondrial activity [146]. Research showed that when DOX is stimulated, the protein expression of SESN2 is drastically decreased [147]. The adverse effects of DOX therapy and the sgRNA deletion of SESN2, which caused mitochondrial dysfunction and cardiomyocyte death by suppressing Parkin-mediated mitophagy, were effectively countered by the injection of SESN2. Mechanistically, SESN2 interacts with p62 and Parkin to facilitate Parkin accumulation in the mitochondria and hinder the suppression of Parkin-mediated mitophagy caused by DOX. After SESN2 overexpression, this process results in the elimination of damaged mitochondria and an improvement in mitochondrial function [78]. The studies have confirmed that SESN2 plays a crucial role in maintaining mitochondrial function and may offer a potential therapeutic solution for DIC.

To better understand therapeutic strategies targeting mitophagy, we categorized them into three groups: Pharmaceutical Drugs, Traditional Medicine, and Potential Targets, as shown in Table 2. Pharmaceutical drugs include compounds that can enhance or inhibit mitophagy, offering versatile treatment options. Traditional medicine provides natural remedies that influence mitochondrial health with fewer side effects. Additionally, identifying potential targets within the mitophagy pathway creates opportunities for developing specific therapies to enhance mitochondrial function. These categories highlight diverse approaches to leveraging mitophagy in therapeutic applications.

**Table 2:** Pharmaceutical treatments and potential non-pharmacological targets used in DOX-induced cardiotoxicity. Chemical compounds mentioned in this article are from PubChem. More specific information is available at the following website: https://pubchem.ncbi.nlm.nih.gov/ (accessed on 10 September 2024).

Name	Chemical Structure	Classification	Model	DOX Treatment	Role in Mitophagy	Cardiac Phenotype	Refs
Vericiguat	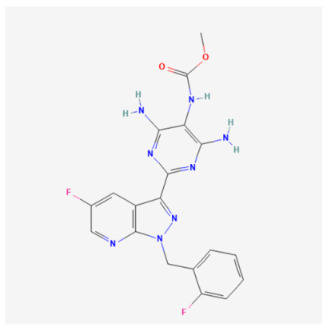	Pharmaceutical drugs	NRCM	1 μM	↑ Mitophagy	↑ Mitochondrial function ↓ Inflammatory factors	[99]
Metformin	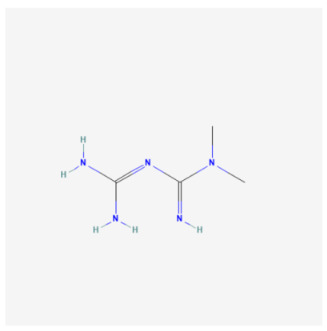	Pharmaceutical drugs	H9C2	1 µM	↓ Mitophagy	↓ Cardiomyocyte death	[103]
Oseltamivir	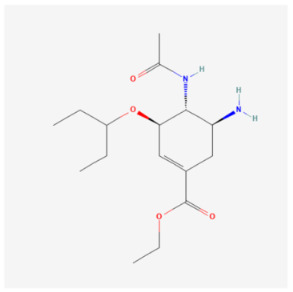	Pharmaceutical drugs	Rat	15 mg/kg	↓ Mitophagy	↑ Cardiac function ↓ Fibrosis ↓ Myocardial injury	[109]
Donepezil	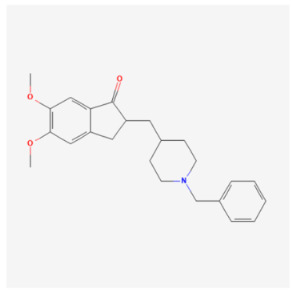	Pharmaceutical drugs	Rat	18 mg/kg	↓ Mitophagy	↑ Cardiac function ↓ Inflammation ↓ Oxidative stress	[113]
SPC	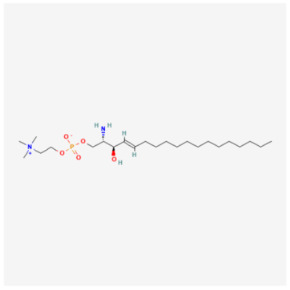	Traditional medicine	Zebrafish	20 μg/g	↓ Mitophagy	↓ Pericardial edema ↓ Myocardial vacuolization ↓ Apoptosis	[117]
Harpagoside	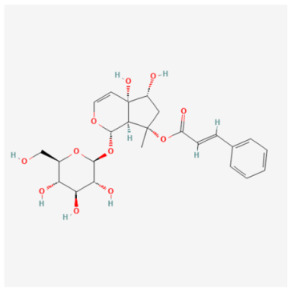	Traditional medicine	Mouse	20 mg/kg	↓ Mitophagy	↑ Cardiac function ↓ Apoptosis ↓ Oxidative stress	[79]
Ellagic acid	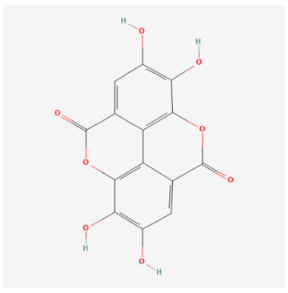	Traditional medicine	NRCM	10 μM	↓ Mitophagy	↓ Cell death	[125]
Berberine	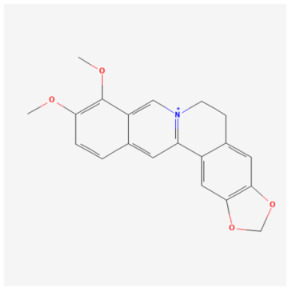	Traditional medicine	Zebrafish	50 μg/g	↑ Mitophagy	↓ Cardiac dysfunction ↓ Cytotoxicity ↓ Apoptosis	[128]
Mesaconine	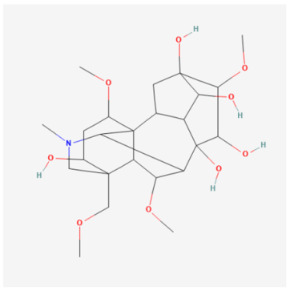	Traditional medicine	Mouse	3.5 mg/kg	↑ Mitophagy	↓ Oxidative stress ↑ Cardiac function	[95]
Rubicon	/	Potential targets	Mouse	20 mg/kg	↑ Mitophagy	↑ Cytoplasmic vacuolization ↑ ROS levels ↓ ATP content	[135]
Mdivi-1	/	Potential targets	Mouse	12mg/kg	↓ Mitophagy	↑ Cardiac function	[137]
MiR-147-y	/	Potential targets	Neonatal pig cardiomyocyte	5 mM	↑ Mitophagy	↓ Apoptosis ↓ Cell death	[141]
IGF-IIR	/	Potential targets	Rat	5 mg/kg	↓ Mitophagy	↑ Apoptosis ↑ Oxidative stress	[142]
SESN2	/	Potential targets	Rat	4 mg/kg	↑ Mitophagy	↓ Apoptosis ↓ Mitochondrial injury ↓ Cardiac dysfunction	[78]

## 6. Problems and Challenges

### 6.1. The Dual Role of Autophagy and Mitophagy

Studies have shown that DOX can activate p53, and the interaction between p53 and Parkin hinders Parkin’s translocation to the outer mitochondrial membrane, thereby inhibiting PINK1/Parkin-mediated mitophagy [148]. This mechanism prevents damaged mitochondria from being removed in a timely manner, leading to cardiotoxicity. On the other hand, DOX has also been found to activate Bnip3-mediated mitophagy [149]. However, this activation may not always be beneficial, as it may lead to the opening of the mPTP and mitochondrial depolarization, which in turn leads to cardiomyocyte death. This dual mechanism of action increases the complexity of mitophagy in DOX cardiotoxicity.

### 6.2. The Complexity of DOX Regulation of Mitophagy

The regulation of mitophagy by DOX involves multiple complex signaling pathways and interactions between key proteins such as TFEB and PGC-1α [150,151]. TFEB may be dephosphorylated and translocated to the nucleus under DOX treatment to promote transcription of autophagy-related genes [152], but the specific mechanism is not fully understood. PGC-1α is involved in mitochondrial biogenesis, energy metabolism, and regulates autophagy [153], but its specific role in DOX cardiotoxicity and its relationship with mitophagy need to be further studied. In addition, there are differences in the sensitivity of different cell types (such as cardiomyocytes and tumor cells) to DOX and the regulatory mechanism of mitophagy [154]. The dose and duration of DOX also significantly affect the regulatory effect of mitophagy, where low doses may not affect mitophagy significantly, while high doses may overactivate or inhibit mitophagy, and the length of treatment will also trigger different cellular responses [155]. Therefore, the regulation of mitophagy by DOX is a complex and multidimensional process.

### 6.3. Contradictory Findings

In DOX studies of mitophagy, the results of different teams are often contradictory. Studies have shown that DOX inhibits mitophagy, reduces autophagy markers, decreases damaged mitochondrial clearance, and increases cardiotoxicity [109,156], possibly by inhibiting TFEB nuclear translocation or downregulating PGC-1α [150,151]. On the contrary, some studies have found that DOX promotes mitophagy, which is manifested by the increase in autophagosomes and the upregulation of PINK1/Parkin expression, which may activate AMPK, Akt/mTOR, and other pathways or directly act on key proteins [148,157]. Currently, there are indeed two distinct and even opposing viewpoints within the research community regarding this matter, and a consensus has yet to be reached. We would like to offer bold hypotheses in light of this ongoing debate. These contradictions may stem from the differences in experimental conditions, the dynamic regulatory characteristics of mitophagy, and the complexity of the regulatory mechanism. Different doses, timings, cell types, and animal strains can all affect results. These propositions aim to bridge the gap between the conflicting perspectives and provide fresh avenues for exploration. We hope to foster a deeper understanding of the issue at hand by examining the underlying mechanisms or conditions that may contribute to these differing outcomes.

## 7. Possible Solutions

### 7.1. Deepen the Study of Mechanisms

In order to thoroughly elucidate the complex mechanism of DOX regulation of mitochondrial autophagy, it is urgent to strengthen basic research and make full use of the power of modern biotechnology. High-throughput sequencing technology can comprehensively unravel the dynamic changes in the transcriptome of cardiomyocytes after DOX treatment [158], revealing which genes are activated or repressed, thus providing clues about the molecular level of mitochondrial autophagy regulation. At the same time, proteomic analysis was able to identify the differences in protein expression profiles before and after DOX treatment [159], especially those proteins directly related to mitophagy, such as TFEB, PGC-1α, PINK1/Parkin, etc., as well as the interaction networks between them. This comprehensive information will help us to construct a complete mechanistic map of DOX regulation of mitophagy.

### 7.2. Consider Multiple Factors

In view of the differences in the regulatory effect of DOX on mitophagy under different experimental conditions, we must fully consider the influence of multiple factors in the research process. First, the specificity of cell types should be clarified, as there may be significant differences in the sensitivity of different cell types to DOX and the regulatory mechanisms of mitophagy. Secondly, the dose and treatment time of DOX should be reasonably set to simulate the actual clinical situation and observe its long-term and short-term effects on mitophagy. In addition, the influence of biological characteristics such as strain, sex, and age of experimental animals on the experimental results should also be considered. Through the comprehensive consideration of multiple factors, we can more comprehensively and accurately evaluate the regulatory effect of DOX on mitophagy and reduce the bias and uncertainty of experimental results.

### 7.3. Carry out Preclinical Validation

Extensive preclinical studies in animal models are a critical step in validating the effectiveness of DOX cardiotoxicity mitigation strategies. By replicating the signs of cardiotoxicity induced by DOX treatment, we can simulate a range of human-like conditions, which allows for the evaluation of various potential interventions such as gene therapy, mitophagy-modifying drugs, and other novel therapeutic approaches. These animal models provide valuable insights into the pathophysiology of DOX-induced cardiotoxicity and help screen out the most promising treatment strategies. Furthermore, they offer essential data that guide the design of subsequent clinical trials, ensuring that the interventions are both safe and effective in human populations. However, it is important to recognize the limitations of animal models in fully recapitulating the complexity of human physiology. Therefore, results from preclinical studies must be interpreted with caution, especially when translating findings to clinical settings. In addition to scientific rigor, preclinical research must adhere to strict ethical guidelines, ensuring that the welfare of the animals is prioritized. This includes minimizing pain and distress, ensuring appropriate care and housing, and conducting studies with well-defined objectives to maximize the scientific value of the experiments. Only by balancing scientific advancement with ethical responsibility can we ensure the reliability and integrity of preclinical research, as well as the eventual success of clinical applications.

### 7.4. Clinical Translational Research

The ultimate goal of research is to apply promising drugs or therapeutic strategies to clinical patients for the regulation of mitophagy. To do this, we need to encourage clinical trials to evaluate the safety and efficacy of these new strategies in humans. Clinical trials should follow scientific and rigorous design principles to ensure the authenticity and reliability of experimental data. At the same time, we should also pay attention to the individual differences and disease complexity of patients so as to provide personalized treatment plans for different patients. Through the validation and optimization of clinical trials, we can translate the results of laboratory research into clinical applications, providing new hope for the treatment of DIC.

### 7.5. Multidisciplinary Collaboration

In the face of the complex challenges of DOX cardiotoxicity, it is particularly important to strengthen multidisciplinary cooperation and communication. Experts in medicine, biology, pharmacy, and other fields should work together to overcome this dilemma. Through interdisciplinary collaboration, we can pool more wisdom and resources to complement each other’s strengths and share resources. At the same time, interdisciplinary collaboration can also promote the generation and application of new ideas and technologies, opening up new avenues for the study and treatment of DOX cardiotoxicity. Therefore, we should actively promote the establishment and improvement of multidisciplinary cooperation mechanisms to contribute more to overcoming the problem of DOX cardiotoxicity.

## 8. Conclusions

Since DOX was first used in clinical settings, its dose-dependent nature and the buildup of hazardous amounts have caused irreversible cardiotoxic side effects, including congestive heart failure and degenerative cardiomyopathy. These side effects have caused serious adverse reactions in many patients receiving cancer chemotherapy, resulting in significant health and economic burden. We review the significant role of mitophagy in DIC and provide an overview of the latest research progress. We also highlight the need for timely monitoring and regulation of mitophagy levels, as well as understanding the factors and mechanisms influencing mitophagy flux. Managing the beneficial effects of mitophagy is crucial for preventing and treating DIC. The PINK1/Parkin, BNIP3/Nix, and FUNDC1 proteins are essential elements of the mitophagy pathway in mammalian cells. These processes are implicated in the development and progression of DIC when mitochondrial function is impaired. There are still unresolved issues that need to be investigated, despite the fact that several medications and targets have emerged and have been demonstrated to reduce DIC through mitophagy. These include figuring out the optimal drug dosage, identifying potential adverse effects, and developing intervention strategies. Even though recent research on mitophagy in DOX cardiotoxicity has produced positive outcomes, it is important to understand that mitophagy’s complete mechanism of action in this condition is still unclear. Thus, in order to provide a solid theoretical foundation and identify practical approaches for the prevention and treatment of DIC in clinical practice, more extensive scientific and clinical research is required.

## Figures and Tables

**Table 1:** Effects of DOX on cardiac mitophagy mechanisms.

Animal Model	DOX Treatment	Cardiac Phenotype	Alternations in Mitophagy	Refs
Mouse	12.5 mg/kg	↓ Cardiac function	↓ Mitophagy	[76]
Mouse	18 mg/kg	↓ Cardiac function ↑ Remodeling	↓ Mitophagy	[77]
Rat	12 mg/kg	↓ Cardiac function ↑ Remodeling ↑ Apoptosis	↓ Mitophagy	[78]
Mouse	20 mg/kg	↓ Cardiac function ↑ Remodeling ↑ Apoptosis	↓ Mitophagy	[79]
Mouse	25 mg/kg	↓ Cardiac function ↑ Remodeling	↑ Mitophagy.	[80]

↓ indicates decrease. ↑ indicates increased.
